# Poorer outcome in stromal HIF-2*α*- and CA9-positive colorectal adenocarcinomas is associated with wild-type *TP53* but not with *BNIP3* promoter hypermethylation or apoptosis

**DOI:** 10.1038/sj.bjc.6604547

**Published:** 2008-08-19

**Authors:** A H G Cleven, B G Wouters, B Schutte, A J G Spiertz, M van Engeland, A P de Bruïne

**Affiliations:** 1 Department of Pathology, GROW – School for Oncology and Developmental Biology, University Hospital Maastricht, PO Box 5800, 6202 AZ Maastricht, The Netherlands; 2 Ontario Cancer Institute/PMH, Toronto, Canada; 3 Department of Molecular Cell Biology, GROW – School for Oncology and Developmental Biology, University Hospital Maastricht, PO Box 5800, 6202 AZ Maastricht, The Netherlands

**Keywords:** colorectal cancer, hypoxia, *TP53*, *BNIP3*, outcome

## Abstract

Stromal expression of hypoxia inducible factor 2*α* (HIF-2*α*) and carbonic anhydrase 9 (CA9) are associated with a poorer prognosis in colorectal cancer (CRC). Tumour cell death, regulated by a hypoxic stromal microenvironment, could be of importance in this respect. Therefore, we correlated apoptosis, *TP53* mutational status and *BNIP3* promoter hypermethylation of CRC cells with HIF-2*α*- and CA9-related poor outcome. In a series of 195 CRCs, *TP53* mutations in exons 5–8 were analysed by direct sequencing, and promoter hypermethylation of *BNIP3* was determined by methylation-specific PCR. Expressions of HIF-2*α*, CA9, p53, BNIP3 and M30 were analysed immunohistochemically. Poorer survival of HIF-2*α* and CA9 stromal-positive CRCs was associated with wild-type *TP53* (*P*=0.001 and *P*=0.0391), but not with *BNIP3* methylation. Furthermore, apoptotic levels were independent of the *TP53* status, but lower in unmethylated *BNIP3* CRCs (*P*=0.004). It appears that wild-type *TP53* in CRC cells favours the progression of tumours expressing markers for hypoxia in their stroma, rather than in the epithelial compartment. Preserved BNIP3 function in CRC cells lowers apoptosis, and may thus be involved in alternative cell death pathways, such as autophagic cell death. However, *BNIP3* silencing in tumour cells does not impact on hypoxia-driven poorer prognosis.

These results suggest that the biology of CRC cells can be modified by alterations in the tumour microenvironment under conditions of tumour hypoxia.

Hypoxia has been reported to influence tumour biology in opposing ways. It can directly induce cell death by activating apoptosis or autophagy, yet hypoxic zones in solid tumours also harbour viable cells resistant to treatment, which contributes to poor patient outcome (Erler *et al*, 2006). Hypoxia influences the expression of genes involved in cell death and energy homeostasis mainly by stabilisation and activation of the hypoxia inducible factor (HIF) family of transcription factors, influencing angiogenesis (*VEGF*), glycolysis (*GLUT1*), pH regulation (*CA9*), apoptosis (*TP53*, *BNIP3*) and autophagy (*BNIP3*) (Bacon and Harris, 2004; Keith and Simon, 2007).

A common hallmark of solid tumours under hypoxic stress is increased ATP requirement, which is supplied by the induction of anaerobic glycolysis. This subsequently leads to a high production of intracellular lactate, requiring regulation of intracellular pH, a process mediated partly by HIF-dependent upregulation of carbonic anhydrase 9 (CA9). Carbonic anhydrase 9 catalyzes the extracellular trapping of acid by hydrating cell-generated CO_2_ into HCO_3_^−^ and H^+^ (Swietach *et al*, 2007). Consequently, CA9 expression may serve as a marker for metabolic adaptation during hypoxia.

Upregulation of p53 and BNIP3 proteins, which stimulate cell death via apoptosis and/or autophagy, appears contradictory to the adverse association between tumour hypoxia and prognosis. However, it was suggested that through induction of cell death, hypoxia selects for cells with defective cell death regulators, such as *TP53* (Graeber *et al*, 1996). In non-selected cells hypoxia can induce expression of p53 and activate p53-mediated G_0_/G_1_ arrest or apoptosis, although secondary effects, such as extracellular acidosis and glucose deprivation, are necessary for p53-mediated apoptosis (Graeber *et al*, 1996; Schmaltz *et al*, 1998; Pan *et al*, 2004; Toledo and Wahl, 2006). Over 50% of human tumours contain somatic mutations in the *TP53* gene, resulting in a defective apoptotic response (Kato *et al*, 2003; Soussi and Lozano, 2005). Therefore, *TP53* mutations are expected to decrease the susceptibility of tumour cells to hypoxia-induced cell death, as shown *in vitro* (Graeber *et al*, 1996; Fei *et al*, 2004). *BNIP3* is a Bcl-2 family member, containing a single BcL-2 homology 3 (BH3) domain and a transmembrane domain localising it to the outer mitochondrial membrane (Vande Velde *et al*, 2000; Lee and Paik, 2006). It is activated by HIF during hypoxia and initiates programmed cell death through apoptosis or autophagy (Ray *et al*, 2000; Mellor and Harris, 2007). Epigenetic silencing of *BNIP3* by promoter hypermethylation has been reported in several cancer types and contributes to resistance to hypoxia-induced cell death (Okami *et al*, 2004; Abe *et al*, 2005; Yan *et al*, 2006). The role of BNIP3 in colorectal cancer (CRC) is unknown, although 66% tumours show *BNIP3* silencing by promoter hypermethylation (Murai *et al*, 2005; Bacon *et al*, 2007).

Usually, the effects of hypoxia in solid tumours are studied within the tumour cells themselves, neglecting the hypoxic response in tumour-associated stroma. In a previous study, we found that hypoxia within the tumour-associated stroma is indeed correlated with a poorer outcome in patients with CRC who are treated by surgery alone. In a multivariate model, stromal expressions of both HIF-2*α* and CA9 were independent adverse prognostic factors, whereas HIF-1*α* was not. Furthermore, expression of hypoxia-related proteins HIF-1*α*, GLUT1 and CA9 in the tumour cells self was not associated with poorer patient survival (Cleven *et al*, 2007).

Our previous findings indicate that hypoxic conditions may modulate the tumour stroma in such a way that a more aggressive tumour behaviour is facilitated, ultimately leading to decreased patient survival.

This study attempts to elucidate whether changes in the epithelial cell compartment of CRC, such as apoptosis and concomitant (epi)genetic changes that are confined to the tumour cells, are related to hypoxia-related changes in the stromal compartment. For this purpose, we correlated alterations of *TP53* and *BNIP3* in tumour cells with expression of hypoxia-related proteins HIF-2*α* and CA9 in relation with patient outcome and apoptotic activity in CRCs.

## Patients and methods

### Patient population

Patients were registered for two multicentre prospective clinical trials in The Netherlands between 1979 and 1981. One trial was designed to compare patient survival after treatment of colon cancer by conventional surgery or the no-touch isolation technique (Wiggers *et al*, 1988). The second trial was conducted to compare survival in rectal cancer patients with or without preoperative radiotherapy. In the current study, we included only the patients who did not undergo preoperative radiotherapy. At the time the trial was conducted, only surgical removal of the tumours was performed, and adjuvant chemotherapy was not yet a standard practice. This study population therefore enables unbiased study of the influence of hypoxic conditions on tumour biology.

Tumour tissues were fixed in buffered formalin, sectioned and embedded in paraffin. Experienced pathologists documented the histopathological characteristics of the tumours (Table 1). Follow-up took place every 3 months during the first 3 years and every 6 months between 3 and 5 years after initial diagnosis and surgery. Standard protocols were followed, with routine blood counts and chemical studies (including CEA levels) at each visit, and liver ultrasound, chest X-ray and colonoscopy annually, to evaluate recurrence of disease and disease-related death. After a 5-year follow-up period, only time and cause of death were registered. Follow-up was complete for all patients. Failure was defined as death due to recurrent disease, excluding postoperative mortality within 30 days and non-disease-related death.

For immunohistochemical and molecular analyses, tumour tissues from 195 CRC patients were available. The distribution of age, gender, tumour stage, location and type of tumour, frequency of events and mean follow-up time of the patients in this study are representative of the patients in the trial (see Table 1).

### Genomic DNA isolation

Genomic DNA was extracted from CRC tissues using PureGene™ genomic DNA isolation kit (Gentra Systems, Minneapolis, MN, USA) based on the manufacturer's protocol.

### TP53 sequencing

Mutation analyses of *TP53* exons 5–8 were performed using a semi-nested PCR approach, (see Supplementary Table 1 for primer sequences). Caco2 (exon 6, codon 204 nonsense mutation) was included as a control. Direct sequencing of PCR products was performed using the BigDye® terminator v1.1 cycle sequencing kit (Applied Biosystems, Foster City, CA, USA) and analysed on the ABI 3730 DNA Analyzer (Applied Biosystems). Mutation was detected using Mutation Surveyor DNA Variant Analysis Software v3.0 (SoftGenetics LLC, State College, PA, USA). The results of the mutation analyses are listed in Table 2. Furthermore, we assessed whether *TP53* missense mutants were transcriptionally active, on the basis of the IARC prediction models (http://p53.iarc.fr/MutationValidationCriteria.asp). Missense mutations were classified as either transactivation-incompetent or transactivation-competent missense mutations.

### BNIP3 promoter methylation analysis

*BNIP3* promoter methylation was determined by sodium bisulphite modification of genomic DNA using the EZ DNA methylation kit (ZYMO Research Co., Orange, CA). Methylation-specific PCR was performed as described in detail elsewhere (Herman *et al*, 1996; Derks *et al*, 2004). DNA was first amplified with *BNIP3* flanking PCR primers that amplify bisulfite-modified DNA but do not preferentially amplify methylated or unmethylated DNA. The resulting template was used as the template for *BNIP3* methylation-specific PCR. For primer sequences see Supplementary Table 1. All PCRs were performed with a control for unmethylated BNIP3 alleles (normal lymphocyte DNA), a positive control for methylated BNIP3 alleles (Sssl methyltransferase (New England Biolabs, Beverly, MA, USA)-treated normal lymphocyte DNA) and a negative control without DNA. Each PCR product was loaded onto a 2% agarose gel, stained with Gelstar® (Cambrex Bioscience Rockland Inc., Rockland, ME, USA) and visualised under UV illumination.

### Immunohistochemistry

Serial formalin-fixed, paraffin-embedded tissues sections (4 *μ*m) were stained for HIF-2*α* and CA9, as described previously (Cleven *et al*, 2007). Brief descriptions are as follows.

#### HIF-2*α* staining

Antigen retrieval was performed by microwave treatment (750 W for 20 min in 1 mM TE buffer, pH 8.0), followed by cooling in buffer for 30 min. Slides were blocked in 25% normal serum for 10 min. Sections were incubated with primary antibody HIF-2*α* (1 : 500) for 100 min (anti-HIF-2*α* monoclonal: ab8365; AbCam, Cambridge, UK).

#### CA9 staining

Slides were blocked in 25% normal serum for 10 min, and then incubated for 45 min with primary CA9 antibody MoAb M75 (1 : 50, anti-human CA9; kindly supplied by Dr S Pastorekova) at room temperature.

In addition to the above-mentioned staining procedures, serial sections were stained for p53, BNIP3 and M30, as follows:

#### p53 staining

Antigen retrieval was performed by microwave treatment (750 W for 15 min in Antigen Retrieval (DAKO, Glostrup, Denmark)), followed by cooling in buffer for 30 min. Slides were blocked in 25% normal serum for 10 min. Sections were incubated for 45 min at room temperature with primary antibody p53 (1 : 500, anti-p53 monoclonal (DO-7); M7001 DAKO).

#### BNIP3 staining

Antigen retrieval was performed by microwave treatment (750 W for 15 min in Antigen Retrieval (DAKO)), followed by cooling in buffer for 30 min. Slides were blocked in 25% normal serum for 10 min. Sections were incubated for 180 min at room temperature with primary antibody BNIP3 (1 : 400, anti-BNIP3 monoclonal (Ana40); ab10433, Abcam, Cambridge, UK).

#### M30 staining

Antigen retrieval was performed by microwave treatment (750 W for 10 min in Antigen Retrieval (DAKO)), followed by cooling in buffer for 30 min. Slides were blocked in 25% normal serum for 10 min. Sections were incubated for 45 min at room temperature with primary Cytodeath antibody M30 (1 : 50, mouse monoclonal (CloneM30); Roche Applied Science, Mannheim, Germany).

Each staining protocol was started with pre-incubating in 0.6% hydrogen peroxide for 20 min to block endogenous peroxidase activity. Furthermore, as a negative control, TBS buffer was used instead of primary antibody. Visualisation was performed using Dako Envision, Peroxidase, mouse System (K4001; DAKO). Powerenvision poly-HRP (50510–60307; Immunologic, Duiven, The Netherlands) was used for M30 visualisation. The slides were counterstained with haematoxylin.

### Evaluation of immunohistochemistry

Evaluation for HIF-2*α* and CA9 staining was performed as described previously in detail (Cleven *et al*, 2007). Briefly, localisation (epithelial or stromal) was scored separately. For the category stromal staining, only the stromal myofibroblasts were taken into account, not the tumour-infiltrating inflammatory cells or the lamina propria of the normal mucosa. If nuclear staining was present in >5% of the tumour epithelial cells or stromal cells, the sample was considered positive for HIF-2*α*.

If membranous staining occurred in >5% of the tumour epithelial cells or stromal cells, samples were considered positive for CA9 (Yoshimura *et al*, 2004).

TP53 and BNIP3 staining were considered positive by the presence of nuclear staining for TP53 and cytoplasmic staining for BNIP3, in >5% of tumour cells.

**Figure 1:**
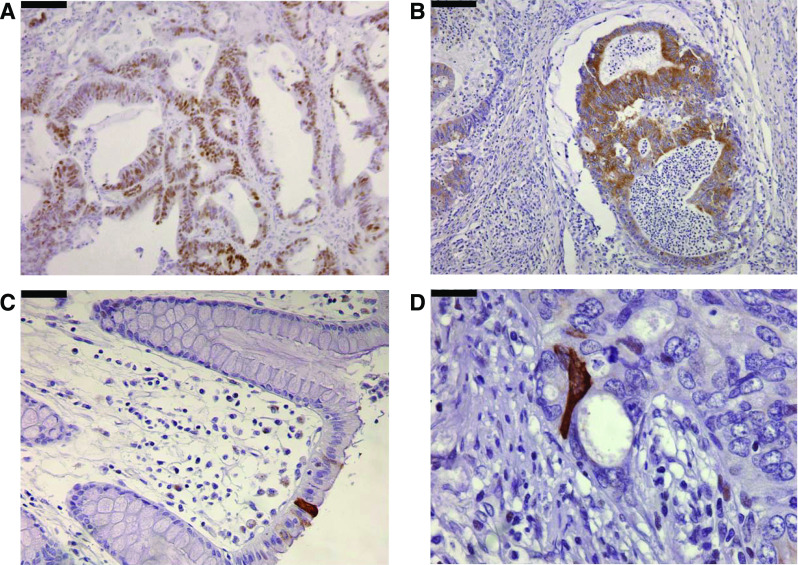
(**A**) Nuclear expression of p53 in colorectal tumour epithelial cells. (**B**) Cytoplasmic expression of BNIP3 in colorectal tumour epithelial cells. (**C, D**) Expression of M30 in colorectal tissue. (**C**) Expression of M30 in normal colon tissue. (**D**) Expression of M30 in tumour epithelial cell. (**A, B**) Original magnification ×20; (**C, D**) original magnification ×40. Black bar pictures: (**A, B**) 200 μm; (**C, D**) 100 μm.

M30 expression was documented as the number of positive M30 cells per square millimetre of tumour cells (counted in 10 high-power fields ( × 100) per tumour) (Figures 1C and D). Apoptosis was categorised as ‘low’ apoptosis when the number of M30-positive cells ⩽10 (mean) and as ‘high’ apoptosis when the number of M30-positive cells >10 (Marijnen *et al*, 2003; de Bruin *et al*, 2006).

### Data analysis

Correlations between HIF-2*α*, CA9, *BNIP3*, *TP53*, M30 and clinicopathological parameters were determined by the Pearson *χ*^2^- and Fisher's exact tests, where appropriate. To evaluate the relationship between HIF-2*α*, *BNIP3*, *TP53* and survival, Kaplan–Meier survival curves were calculated. Differences between groups were determined by using the Log-rank test. The end point for analyses was overall survival starting from the day of surgery. All *P*-values are two sided and *P*<0.05 was considered statistically significant. Correction for multiple comparisons was performed using the Bonferroni procedure. Patients with unknown and unspecified scores have been omitted from analyses for that specific variable. SPSS 12.0 software was used for data analyses.

## Results

### *TP53* mutations

*TP53* mutation analysis was successful in 155 out of 195 (79%) CRCs. Out of the 155 CRCs, 72 (46%) were classified as having no *TP53* mutations, 4 (3%) as having silent mutations and 2 (1%) as having known common polymorphism (exon 6, codon 213, CGA>CGG, R/R, refSNP rs1800372) (Table 2). A total of 37% (57 out of 155) CRCs were classified as transactivation-incompetent missense mutations and 5% (8 out of 155) CRCs as transactivation-competent missense mutations. Correlations between *TP53* and other variables did not change with respect to the predicted presence or absence of transcriptional activity of *TP53* mutants. Therefore, in further analyses, CRCs were classified as *TP53* wild type when no mutations, silent mutations or a known common polymorphism were found, and as mutant *TP53* when CRCs had either missense, nonsense or frame-shift mutations. Using this classification, 77 out of 155 (50%) CRCs showed *TP53* mutations (Table 1), which is in agreement with data published by others (Cripps *et al*, 1994; Pricolo *et al*, 1997; Borresen-Dale *et al*, 1998; Kressner *et al*, 1999; Tortola *et al*, 1999; Garrity *et al*, 2004; Tang *et al*, 2004; Conlin *et al*, 2005; Mollevi *et al*, 2007; Petitjean *et al*, 2007).

p53 protein expression was only observed in the nucleus of epithelial cells (Figure 1A). There was a significant correlation between the absence of p53 protein expression and wild-type *TP53 vs* the presence of p53 protein in mutant *TP53* (*P*=0.029; data not shown). No correlation was observed between the *TP53* mutation status and clinicopathological data (Table 1).

### *TP53* mutations, patient survival and apoptosis

**Figure 2:**
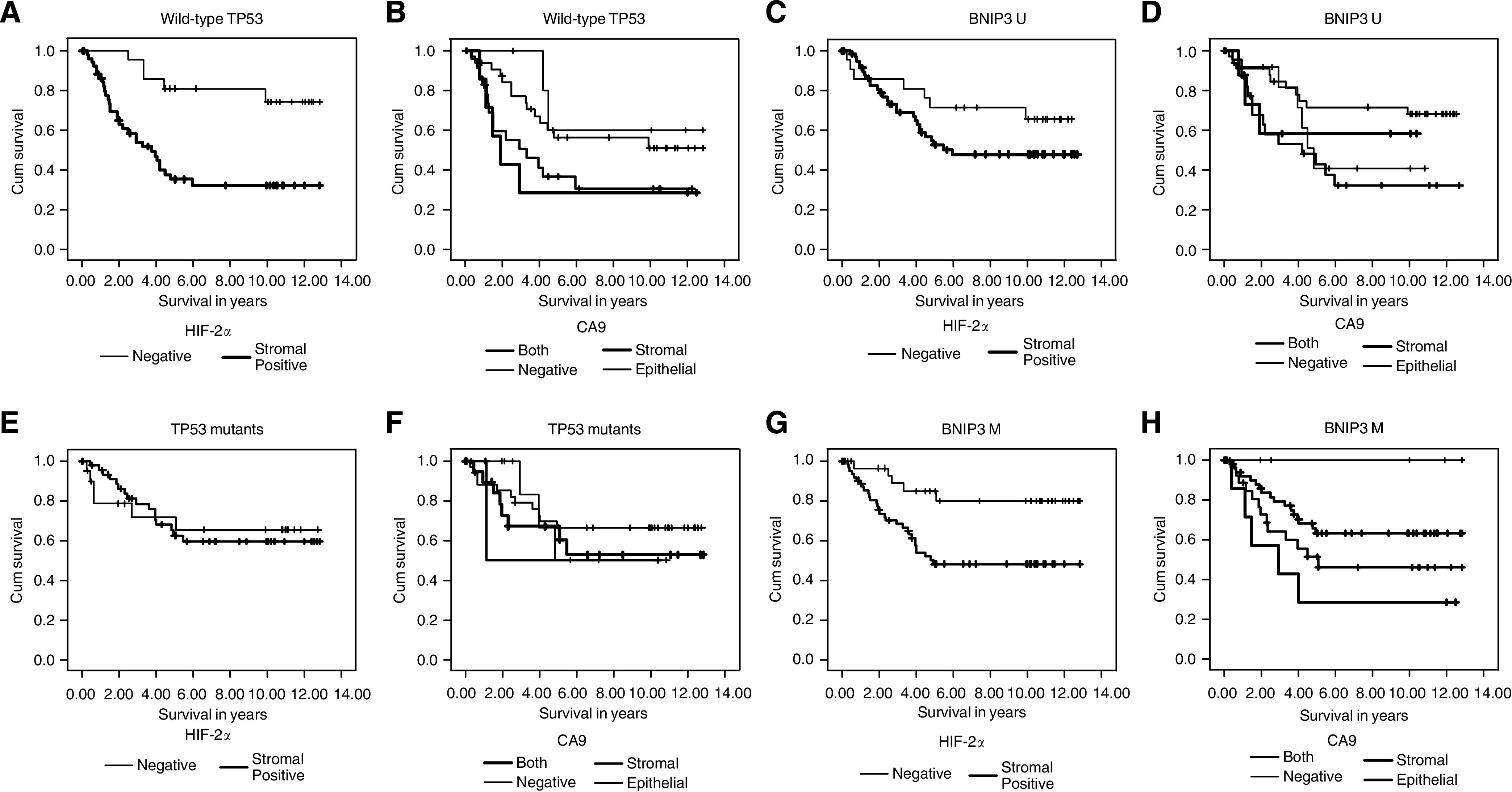
Survival curves. (**A**) HIF-2*α* expression in wild-type *TP53* group (*P*=0.001, *n*=75). (**B**) CA9 expression in wild-type *TP53* group (*P*=0.0829, *n*=72). (**C**) HIF-2*α* expression in *BNIP3* unmethylated group (*P*=0.1712, *n*=83). (**D**) CA9 expression in *BNIP3* unmethylated group (*P*=0.0725, *n*=79). (**E**) HIF-2*α* expression in mutant *TP53* group (*P*=0.9312, *n*=69). (**F**) CA9 expression in mutant *TP53* group (*P*=0.8456, *n*=71). (**G**) HIF-2*α* expression in *BNIP3* methylated group (*P*=0.006, *n*=98). (**H**) CA9 expression in *BNIP3* methylated group (*P*=0.0495, *n*=95). U, unmethylated; M, methylated.

Overall, no significant survival difference was observed between wild-type and mutant *TP53* CRCs (data not shown). However, the previously reported association between HIF-2*α*- or CA9-positive CRCs and poor prognosis was found to exist exclusively in wild-type *TP53* CRCs (*P*=0.001 and *P*=0.0829, respectively; Figures 2A and B). Furthermore, there was a significant difference in survival between stromal CA9 expression (38%, 5-year survival) and epithelial CA9 expression (71%, 5-year survival) within wild-type *TP53* tumours (*P*=0.0391, data not shown). Overall levels of HIF-2*α* or CA9 expression were not different between wild-type and mutant *TP53* CRCs (data not shown). Survival of mutant *TP53* CRCs was not related to HIF-2*α* or CA9 expression (*P*=0.9312 and *P*=0.8456, respectively; Figures 2E and F). These data suggest that wild-type *TP53* CRCs are less susceptible to the adverse effects of hypoxia. As *TP53* can induce apoptosis during hypoxia, we assessed the extent of apoptosis (M30 staining; Figures 1C and D). Overall, we found no differences in apoptotic levels between wild-type and mutant *TP53* CRCs (Table 3), or between HIF-2*α*- and CA9-positive or -negative CRCs (data not shown). This was found regardless of the *TP53* mutation status. These results indicate that the presence or absence of functional p53 protein is not decisive for determining the extent of apoptosis in CRCs.

### *BNIP3* methylation

*BNIP3* promoter methylation analysis was successful in all of the 195 CRCs (100%). Overall, 53% (103 out of 195) CRCs showed *BNIP3* promoter hypermethylation (Table 1), which is in agreement with data published earlier (Murai *et al*, 2005; Lee and Paik, 2006).

The relationship between protein expression of BNIP3 and *BNIP3* promoter methylation status was analysed in a randomly selected subset of patients (*n*=31). BNIP3 protein expression was only observed in the cytoplasm of epithelial cells (Figure 1B). *BNIP3* promoter-methylated CRCs less frequently demonstrated BNIP3 protein expression than unmethylated CRCs (25 *vs* 75%, respectively).

### *BNIP3* methylation, patient survival and apoptosis

Overall, there was no significant survival difference between *BNIP3* methylated and unmethylated CRCs (data not shown). Although in HIF-2*α* stromal-negative CRCs, *BNIP3* methylation occurred in 61% (33 out of 54) and did not influence prognosis, in the HIF-2*α*-positive tumours, methylation was observed at almost equal frequency, 52% (68 out of 132), but was associated with poorer patient survival (*P*=0.006; Figure 2G). Similarly, exclusively stromal (and not epithelial) expression of CA9 was an indicator of a poorer prognosis in both *BNIP3* methylated and unmethylated tumours (*P*=0.0495 and *P*=0.0725, respectively; Figures 2D and H).

This suggests that hypoxic CRCs with stromal expression of HIF-2*α* and CA9 have a poorer prognosis, independent of *BNIP3* methylation.

As *BNIP3* has been reported to induce apoptosis in response to hypoxia, its methylation and associated downregulation might be expected to result in less apoptosis in the HIF-2*α* subgroup. However, a low apoptotic activity (low M30 expression) was detected more frequently in *BNIP3* unmethylated CRCs 68% (50 out of 73) compared with *BNIP3* methylated CRCs 46% (42 out of 91, *P*=0.004; Table 3). Although tumours with both methylated *BNIP3* and stromal HIF-2*α* expression showed a poorer patient survival when compared with HIF-2*α*-negative tumours, this was not related to apoptosis (data not shown). Furthermore, we did not detect differences in apoptotic levels between tumours with or without CA9 expression, regardless of the *BNIP3* methylation status.

## Discussion

In a previous study on the expression of hypoxia-related markers (HIF1*α*, HIF2*α*, CA9 and GLUT1) in colorectal adenocarcinomas, we found that in all tumours at least one of these proteins is immunohistochemically expressed. This indicates that hypoxia is an all but ubiquitous phenomenon in colorectal tumours. However, expression of only HIF2*α* and CA9 in the tumour-associated stroma was correlated with a poorer prognosis, suggesting that tumours with this particular phenotype follow a more aggressive course (Cleven *et al*, 2007). These results are in contrast to some other studies on colon and rectum tumours that can be summarised by the observation that HIF1*α* and GLUT1 expression in rectal cancer cells is of prognostic significance (Haber *et al*, 1998; Yoshimura *et al*, 2004; Lu *et al*, 2006; Theodoropoulos *et al*, 2006). Our finding was relatively new, in the sense that others have mainly reported the relation between tumour prognosis and expression of hypoxia markers in cancer cells, without paying attention to stromal expression. The results in our study did not differ between tumours of the colon and rectum.

So far, the biological basis of stromal expression of hypoxia-related markers in CRC is unclear. It could either be a serendipitous finding, or a genuine indication of altered epithelial–mesenchymal interactions within a subset of tumours. In these tumours, hypoxia-driven metabolic and cell biological changes could hypothetically alter the tumour stroma toward an environment that can facilitate cancer progression, along multiple routes, leading to an enhanced proliferation or survival of tumour epithelial cells (Beppu *et al*, 2008). Under hypoxic conditions, the tumour stroma can select for the propagation of certain subclones of cancer cells that are optimally endowed for tumour progression. A recent study on breast cancer, on how changes in stromal gene expression affect epithelial tumour progression, showed that gene expression profiling of microdissected tumour stroma resulted in a set of stromal genes, which could predict clinical outcome. This set notably included enhanced stromal expression of hypoxia-associated genes (Finak *et al*, 2008).

The current study attempts to pinpoint specific genetic and epigenetic features of CRC cells, which are both associated with the observed more aggressive presentation of CRCs expressing HIF2*α* and CA9 in their surrounding stroma, and impact on one of the hallmarks of cancer, namely regulation of apoptosis under hypoxic conditions.

Tumour hypoxia results in the induction of pro-death signals, mediated partly by *TP53* and *BNIP3* (Brahimi-Horn and Pouyssegur, 2006). Therefore, it could be envisaged that hypoxia provides a selective environment for outgrowth of cells in which these genes have become mutated or silenced. Loss of pro-death genes, such as *TP53* and *BNIP3*, may result in increased hypoxia tolerance and cross-resistance to other death-inducing stimuli associated with metabolic stress or treatment. Immunohistochemical staining showed that *TP53* and *BNIP3* expressions are confined to the epithelial cell compartment in CRC, and are not present in the surrounding mesenchymal cells, making these two genes interesting candidates to study the hypothesis of cancer cell selection under hypoxia-driven modification of tumour–stroma interactions.

Intriguingly, our results indicate that tumours expressing HIF-2*α* or CA9 in their stroma have a poorer prognosis in wild-type *TP53* tumours compared with mutant tumours. It is unclear as to how wild-type *TP53* might benefit this tumour subgroup, but several possibilities exist. Firstly, p53 is involved in a metabolic switch to glycolysis when oxidative phosphorylation is impaired during hypoxia (Lum *et al*, 2007). Also, other means of adaptation to metabolic stress, such as increased fatty acid *β* oxidation, have been shown to be present in tumour cells with intact p53. With respect to this, increased apoptosis was reported in p53-deficient HCT-116 CRC cells as compared with wild-type p53 HCT-116 cells, when challenged by metabolic stress (Buzzai *et al*, 2007). The second possibility is that wild-type *TP53* does not act directly, but simply correlates with defects in another pathway, such as the *BNIP3* cell death pathway, which substitutes for *TP53* loss in a similar fashion during carcinogenesis.

With respect to apoptosis- and hypoxia-driven tumour progression, we did not find important effects related to the mutational status of *TP53.* However, apoptotic levels were lower in BNIP3-expressing tumours, when compared with tumours with epigenetically silenced BNIP3 (*P*=0.004), which is somewhat surprising given the fact that functional BNIP3 is thought to induce cell death downstream of hypoxia inducible transcription factors. Apparently, things are more complicated. BNIP3 may not be restricted to regulation of apoptosis, but could also regulate other pathways, such as autophagy, in which there is a delicate balance between cell survival and cell death (Papandreou *et al*, 2005). Conceivably, the lower apoptotic activity in tumours with functional BNIP3 might be due to autophagic rescue of the tumour cells. Furthermore, BNIP3 levels appear to modulate cell death not only via apoptosis or autophagy, but also via necrosis. Also, the net effect of BNIP3 is determined by the level of expression: too high BNIP3 expression will lead to autophagic cell death, whereas lower levels of BNIP3 expression, as in cells where *BNIP3* is silenced, will induce necrosis (Tracy *et al*, 2007). In our study, we used the immunohistochemical marker M30, which exclusively measures apoptotic cell death, and thus were not able to differentiate between other forms of cell death, such as autophagic death and necrosis (Leers *et al*, 1999; Ueno *et al*, 2005).

Summarising the results from the current study, levels of apoptosis do not play an important role in determining the poorer prognosis of hypoxic CRCs, as defined by stromal expression of HIF-2*α* and CA9. However, the latter phenotype is correlated with the presence of wild-type *TP53* in the tumour cells, and the presence of functional p53 does indeed appear to have an important impact on poorer prognosis. This prognostic effect is not established through regulation of programmed tumour cell death, but may rather be connected to an enhanced capacity for adequate adaptation to metabolic stress. As *TP53* mutations occur in a relatively early stage of colorectal carcinogenesis, the potential deleterious effects of hypoxia on CRC biology may already be programmed in a very early phase of tumour development.

As opposed to *TP53*, functionally or epigenetically silenced *BNIP3* did not turn out to be of influence in determining tumour prognosis. Furthermore, preservation of *BNIP3* function was shown to decrease apoptotic activity, and may thus be involved in enhanced cell survival through autophagic rescue, or could be implicated in alternative cell death pathways, such as autophagic cell death or necrosis, which we were unable to measure in our experimental approach.

The findings in this translational study, on the relation between expression patterns of hypoxia-related markers in clinical samples of CRC and the functional status of genetically or epigenetically modified proteins involved in regulation of tumour cell death on the one hand and patient outcome on the other, open up interesting new avenues for more fundamental studies on the mechanisms underlying tumour hypoxia-induced changes in epithelial–mesenchymal interactions.

Understanding the mechanisms by which hypoxic tumours can overcome cell death signals and adapt through metabolic changes is critical for our understanding of tumour progression and development of effective therapeutics in CRC patients with adverse prognostic profiles.

## Figures and Tables

**Table 1:** *TP53* mutation analyses, *BNIP3* methylation and clinicopathological characteristics

	***TP53***				***BNIP3***					
	**Wt**		**Mutant**			**U**		**M**		
	**No.**	**%**	**No.**	**%**	***P***	**No.**	**%**	**No.**	**%**	***P***
Total patients	78	(50)	77	(50)		92	(47)	103	(53)	
										
*Age (years)*					0.297					0.759
<69	35	(45)	41	(53)		44	(48)	47	(46)	
>69	43	(55)	36	(47)		48	(52)	56	(54)	
										
*Sex*					0.942					0.788
Male	35	(45)	35	(45)		42	(46)	49	(48)	
Female	43	(55)	42	(55)		50	(54)	54	(52)	
										
*Tumour location*					0.202					0.016
Proximal	32	(41)	24	(31)		25	(27)	45	(44)	
Distal	46	(59)	53	(69)		67	(73)	58	(56)	
										
*Tumour*					0.819					0.967
Colon	49	(63)	47	(61)		61	(66)	68	(66)	
Rectum	29	(37)	30	(39)		31	(34)	35	(34)	
										
*Differentiation*					0.913					0.959
Well	8	(10)	8	(10)		10	(11)	11	(11)	
Moderate/ poor	70	(90)	69	(90)		82	(89)	92	(89)	
										
*TNM*					0.471					0.001
1	1	(1)	2	(2)		3	(4)	1	(1)	
2	40	(51)	46	(60)		61	(66)	55	(53)	
3	28	(36)	19	(25)		14	(15)	40	(39)	
4	9	(12)	10	(13)		14	(15)	7	(7)	

Wt=wild type; U=unmethylated; M=methylated; TNM=tumour, node, metastasis.

Location: proximal or distal to splenic flexure.

**Table 2:** Frequency and type of *TP53* alterations

***TP53* Mutation**	**No.**	**%**
Wild-type	72	(46)
Tr-incom missense	57	(37)
Tr-com missense	8	(5)
Silent	4	(3)
Nonsense	5	(3)
Deletion	4	(3)
Insertion	3	(2)
Polymorphism	2	(1)
Total	155	(100)

Tr-incom=transactivation incompetent; Tr-com=transactivation competent.

**Table 3:** Correlations between *BNIP3* methylation and *TP53* alteration *vs* M30 expression

	**M30 low**		**M30 high**		
	**No.**	**%**	**No.**	**%**	**P**
*BNIP3*					
Unmethylated	50	(68)	23	(32)	0.004
Methylated	42	(46)	49	(54)	
					
*TP53*					
Wild-type	39	(56)	31	(44)	0.703
Mutation	36	(59)	25	(41)	
