# Supplementary material for: Poorer outcome in stromal HIF-2α- and CA9-positive colorectal adenocarcinomas is associated with wild-type TP53 but not with BNIP3 promoter hypermethylation or apoptosis
Source: Br J Cancer. 2008 Aug 19;99(5):727–33. doi: 10.1038/sj.bjc.6604547 (PMC2528150; doi:10.1038/sj.bjc.6604547)
Supplement: Supplementary Table 1 [file 6604547x1.doc]

| *TP53* | Primer | Sequence | Product size |
| --- | --- | --- | --- |
| 5 exon | Flank sense  Flank antisense  Inside sense | 5’ CTG TTC ACT TGT GCC CTG AC 3’  5’ AGC CCT GTC GTC TCT CCA G 3’  5’ TGC CCT GAC TTT CAA CTC TG 3’ | 268 bp  257 bp |
| 6 exon | Flank sense  Flank antisense  Inside antisense | 5’ GGC CTC TGA TTC CTC ACT G 3’  5’ ATA AGC AGC AGG AGA AAG CC 3’  5’ GAC CCC AGT TGC AAA CCA G 3’ | 240 bp  164 bp |
| 7 exon | Flank sense  Flank antisense  Inside antisense | 5’ CAT CTT GGG CCT GTG TTA TC 3’  5’ GAT GTG ATG AGA GGT GGA TGG 3’  5’ GGA AGA AAT CGG TAA GAG GTG 3’ | 261 bp  230 bp |
| 8 exon | Flank sense  Flank antisense  Inside antisense | 5’ GAT TTC CTT ACT GCC TCT TGC 3’  5’ CAT AAC TGC ACC CTT GGT CTC 3’  5’ TCC TCC ACC GCT TCT TGT C 3’ | 231 bp  212 bp |
| *BNIP3* | Primer | Sequence | Product size |
|  | Flank Sense  Flank  antisense | 5’-TTY GTT TTG TTT TGT GAG TTT TTT-3’  5’-CCR AAC TAC AAA ATA TAC TTC AAC TAC-3’ | 129 bp |
|  | Unmethylated sense  Unmethylated antisense | 5’-GGT TTT GTT TAG TTT GGG AGT G-3’  5’-CCT CAA CTA CAA ACA ATA AAA AAA CA-3’ | 74 bp |
|  | Methylated sense  Methylated antisense | 5’-TTT CGT TTA GTT CGG GAG CG-3’  5’-CAA CTA CGA ACG ATA AAA AAA CG-3’ | 69 bp |

**Supplement Table 1**: *TP53* mutation analysis primers and *BNIP3* MSP primer sequences
